# Synergistic impacts by an invasive amphipod and an invasive fish explain native gammarid extinction

**DOI:** 10.1186/s12898-016-0088-6

**Published:** 2016-07-14

**Authors:** S. Beggel, J. Brandner, A. F. Cerwenka, J. Geist

**Affiliations:** Aquatic Systems Biology Unit, School of Life Sciences Weihenstephan, Technical University of Munich, 85354 Freising, Germany; Wasserwirtschaftsamt Regensburg, Kavalleriestraße 2, 93053 Regensburg, Germany

**Keywords:** *Dikerogammarus villosus*, *Gammarus pulex*, *Neogobius melanostomus*, Selective predation, Anti-predator behaviour, Species displacement

## Abstract

**Background:**

Worldwide freshwater ecosystems are increasingly affected by invasive alien species. In particular, Ponto-Caspian gobiid fishes and amphipods are suspected to have pronounced effects on aquatic food webs. However, there is a lack of systematic studies mechanistically testing the potential synergistic effects of invasive species on native fauna. In this study we investigated the interrelations between the invasive amphipod *Dikerogammarus villosus* and the invasive fish species *Neogobius melanostomus* in their effects on the native amphipod *Gammarus pulex*. We hypothesized selective predation by the fish as a driver for displacement of native species resulting in potential extinction of *G. pulex*. The survival of *G. pulex* in the presence of *N. melanostomus* in relation to the presence of *D. villosus* and availability of shelter was analyzed in the context of behavioural differences between the amphipod species.

**Results:**

*Gammarus pulex* had a significantly higher susceptibility to predation by *N. melanostomus* compared to *D. villosus* in all experiments, suggesting preferential predation by this fish on native gammarids. Furthermore, the presence of *D. villosus* significantly increased the vulnerability of *G. pulex* to fish predation. Habitat structure was an important factor for swimming activity of amphipods and their mortality, resulting in a threefold decrease in amphipods consumed with shelter habitat structures provided. Behavioral differences in swimming activity were additionally responsible for higher predation rates on *G. pulex*. Intraguild predation could be neglected within short experimental durations.

**Conclusions:**

The results of this study provide evidence for synergistic effects of the two invasive Ponto-Caspian species on the native amphipod as an underlying process of species displacements during invasion processes. Prey behaviour and monotonous habitat structures additionally contribute to the decline of the native gammarid fauna in the upper Danube River and elsewhere.

## Background

Worldwide freshwater ecosystems are undergoing major changes in biodiversity, mainly caused by anthropogenic habitat modification and biological invasions [1, 2]. Alteration of habitat and ship traffic are known to be mainly responsible for the dispersal of invasive alien species (IAS) in aquatic ecosystems. Transportation vessels are vectors for introduction of IAS via ballast water or ship-hull transfer from their origins to new areas [3]. In case of successful introduction, the establishment of IAS can result in significant declines of native taxa. Channelized rivers thereby provide both suitable habitat structures [4] as well as migration corridors by interconnecting catchments and enabling dispersal of non-native species [5].

Over the last two decades, ongoing invasions, especially by Ponto-Caspian crustaceans, molluscs and fishes have been reported from the middle and upper sections of the Danube River [6–9], the Rhine River [10, 11] and other parts of the world [12, 13].

Among these successful invaders, the amphipod *Dikerogammarus villosus* (Sovinskij 1894) has frequently been proposed to affect native amphipod populations worldwide [5, 14–18]. Corresponding to its first records in the German sections of the Danube River in 1992 and the Rhine River in 1995 [19], significant decreases in abundance and distribution of indigenous amphipods such as *Gammarus pulex* L. 1758 and *Gammarus roeselii* Gervais 1835 have been observed [16, 20, 21], yet these declines have not been mechanistically linked to the simultaneous increase in the abundance of invasive species. Several recent studies identified functional feeding responses and asymmetric mutual predation of *D. villosus* as important mechanisms probably facilitating competitive advantages over other amphipods [21, 22]. Previous studies focused on selective predation of native and invasive amphipods by fishes, e.g., rainbow trout *Oncorhynchus mykiss*, European perch *Perca fluviatilis* [23], and burbot *Lota lota* [24]. Since these fishes only occur in low abundances and in certain areas of the upper Danube River, they can be ruled out as an explanation for the massive declines of native amphipod populations in this river section.

About one decade after the introduction of *D. villosus*, the round goby *Neogobius melanostomus* (Pallas 1814) arrived in the German section of the Danube River in 2004 [25], rapidly spread and displayed high levels of population differentiation [26, 27]. Within few years, this goby species strongly increased in abundance, currently contributing more than three quarters of abundance and about two thirds of biomass of fish in artificial rip-rap bank habitats [7, 8]. Its range expansion and population growth is still ongoing. Extensive samplings during the years 2010 to 2011 [7, 8] were no longer able to detect the formerly abundant native amphipod species, *G. pulex* and *Gammarus roeseli*, in the upper Danube River. Instead they only found the non-native *D. villosus* as the currently most abundant and widely distributed amphipod. Consequently, declines to extinction of native amphipods in the upper Danube River could have been influenced by the invasion of *N. melanostomus*, particularly since amphipods were identified as most important prey for this fish in different field surveys [7, 28, 29].

Since synergistic effects of multiple invasive species can potentially accelerate biodiversity loss and may enforce further homogenization of biological communities, the term “invasional meltdown” has been proposed for such interaction on an ecosystem scale [30]. However, to our knowledge, no study systematically analysed mechanisms and potential  sympatric impact of *N. melanostomus* and *D. villosus* on indigenous amphipods in experimental trials, to validate species interaction in native species displacement processes.

Invasional processes in general cannot be captured within a short time-frame and the assessment of possible negative consequences for a respective system can be rather complex. However, investigations of species interactions under controlled conditions have the potential to reveal mechanistic relationships that can support the holistic understanding of complex invasion processes.

In this study, selective predation of *N. melanostomus* on native and non-native amphipods was investigated in controlled experiments. The main goals of the present study were (i) to estimate predation preference of *N. melanostomus* towards one of the respective amphipod species, (ii) to determine potential interaction between invasive *D. villosus* and native *G. pulex*, and (iii) to test how potential interactions affect predation by *N. melanostomus*, as synergistic impact in native species displacement.

We hypothesized that (i) there is a feeding preference in *N. melanostomus* towards indigenous amphipod species explaining their massive declines in nature, and that (ii) *D. villosus* can increase the relative predation risk of *G. pulex* by interference competition and a more effective predator avoidance due to the sympatric origin with *N. melanostomus.*

## Methods

### Test species

*Neogobius melanostomus* were collected by electrofishing in the upper Danube River (Bad Abbach, Germany: N48°57′11.56″, E11°59′12.53″) under license number 31-7562. The collection of gobies was additionally approved by the local owner of the fisheries rights (Kreisfischereiverein Kelheim e.V.) and the fisheries authority (Fischereifachberatung Niederbayern). Specimens with a mean total length (*L*_T_) of 10.0 ± 2.0 cm were used since this size class is known to preferentially feed on amphipods [7]. After transportation to the laboratory, fish were acclimatised in aerated aquaria (100 × 40 × 50 cm, density of 20 to 30 fish/m^2^, each equipped with 10 clay tubes as shelter) for 1 week prior to the experiments. During the acclimatisation period fish were fed with commercial fish-food (trout chow, Skretting, Norway). Invasive *D. villosus* were collected in the upper Danube River near Kelheim, Germany (N48°54′56.61″, E11°51′43.80″). Since no *G. pulex* were found in the main channel of the Danube River in recent years, we collected them from a small headwater tributary of the Danube River system, the Moosach River (Freising, Germany: N48°23′38.83‘‘, E11°43′26.15″). Accordingly, the native amphipods were completely “naïve” to the other test species. After kick-sampling, amphipods were sorted into two size classes (by sieving with mesh sizes of 2 and 1.5 mm) and transported to the laboratory in an aerated cooler. The smaller size class (size class 1) had a mean *L*_T_ ± SD of 11.8 ± 1.5 mm for *G. pulex* and 12.0 ± 1.4 mm for *D. villosus*, (measured on random samples of n = 30 per species and size class).The larger size class (size class 2) in both species had a mean *L*_T_ ± SD of 14.8 ± 1.3 mm for *G. pulex* and 18.3 ± 2.4 mm for *D. villosus*. Both amphipod species were acclimatised to laboratory conditions in separated aerated aquaria (60 × 30 × 30 cm with coarse pebbles as substratum, resembling their natural shelter) for 24–48 h. During the acclimatisation period, amphipods were fed ad libitum with pre-conditioned black alder-leaves (*Alnus glutinosa*) and fish-food (trout chow, Skretting, Norway). Test species collection and experimental testing was conducted in August and September 2012.

### Test conditions

Experimental trials were conducted under constant physicochemical conditions (mean ± SD; temperature (T)12.3 °C ± 0.5 °C, dissolved oxygen (DO) 8.9 ± 1.2 mg L^−1^, electric conductivity (EC, at 25 °C) 1125 ± 5 µS cm^−1^ using local well water. Ionic composition of the water is given in Table 1. Light conditions were 12:12 h dark:light. The test setup consisted of 14 aquaria (40 × 25 × 25 cm) individually supplied with a constant water flow(-through) of 0.8 L min^−1^. Adjacent aquaria were shielded from each other to avoid learning effects between the test organisms of different treatments and to avoid mutual disturbance. Each aquarium was filled with 1.5 L of pebbles (16–32 mm) as substratum (“substratum present”) or kept without substratum (“substratum absent”) and equipped with a clay tube providing shelter for the goby (open side facing back wall). All experimental trials were conducted consequently under daylight conditions to minimize variation due to diel changes in the organisms’ behaviour [13, 31]. The experiments were conducted according to German Tierschutzgesetz (§11 TierSchG), approved by the local veterinary board (Landratsamt Freising, license number 32-568) and the animal welfare committee at TUM.

**Table 1:** Water chemistry parameters

Parameter	Concentration [mg L^−1^]
Sodium (Na^+^)	42.3
Potassium (K^+^)	9.6
Calcium (Ca^2+^)	123
Magnesium (Mg^2+^)	41.6
Iron (Fe^3+^)	<0.1
Manganese (Mn^2+^)	<0.05
Chloride (Cl^−^)	130
Bromide (Br^−^)	<0.05
Hydrogen carbonate (HCO_3_^−^)	311
Sulfate (SO_4_^2−^)	58
Nitrate (NO_3_^−^)	10.4
Ortho-phosphate (PO_4_^3−^)	0.09
Ammonium (NH_4_^+^)	<0.02
Dissolved organic carbon (DOC)	0.94
Total inorganic carbon (TIC)	70.6

### Preliminary experiments

The optimum duration of the feeding trial was determined by preliminary experiments that were not included in the statistical analyses. Duration of 3 h was identified to be most suitable for the quantification of consumption rates (20.7 % ± 9 SD), based on the criteria that 100 % of the fish were feeding and gut contents were not fully digested to allow prey identification.

### Amphipod mortality without predator

Several experimental trials were conducted to test for gammarid mortality without the presence of *N. melanostomus* caused by random mortality or intraguild predation (IGP, [32]). Each experimental trial consisted of 15 (single species trial) or 30 (mixed species trial) replicates, respectively. Each replicate was stocked with a total of 40 individuals, either 40 specimens of the same species (single species trial), or 20 specimens from both species (mixed species trial). In mixed species trials, two different experimental approaches were conducted: On the one hand, *G. pulex* and *D. villosus* of the same size class (size class 1 as described above) were used. In addition, *D. villosus* individuals of size class 2 vs. *G. pulex* of size class 1 were exposed to account for the different maximum sizes of the two species. Test duration of each trial was 3 h.

### Predation experiments

Experimental trials were conducted in absence and in presence of substratum. Predation experimental trials consisted of 11 (single species trial) or 22 (mixed species trial) replicates without substratum, respectively. In experiments with substratum added, a higher number of replicates was used in the single species trials (*G. pulex*: 49; *D. villosus*: 14) due to the greater expected behavioural variability in these treatments with more complex habitat structure. Aquaria were stocked with 40 individuals of the same amphipod species (single species trial) or 20 each from both species (mixed species trial). In mixed species trials, either *G. pulex* and *D. villosus* of the same size class (size class 1) were used or size class 2 *D. villosus* individuals vs. size class 1 *G. pulex* to account for naturally expected body size-dependent effects. Predation experiments for different size classes were conducted as mixed species trial.

Gobies were not fed 24 h prior to the experiments. Amphipods transferred to the test aquaria were allowed to settle and shelter for 1 h before fish were added. Test duration of each trial was 3 h. Within this timeframe, fish exhibited natural behaviour and consumed amphipods, as expected from preliminary experiments. These short-time experiments allowed for an easy assignment of amphipod species identities and numbers in subsequent gut-content analyses, since prey was nearly undigested. After each feeding trial, fish were caught with a dip-net, euthanized and immediately frozen at −20 °C. The remaining living and dead amphipods in each aquarium were counted. Prior to dissection for stomach content analysis, *L*_T_ of the fish (to the nearest 1 mm) and total body mass (*W*_T_ to the nearest 0.001 g) were measured. Sex of the fish was determined using the morphology of the urogenital papilla according to Kornis et al. [13] before the experiments and later double-checked during dissection. Intestinal tracts (from the pharyngeal teeth to the anus) were removed, full and empty wet-weight was measured (nearest 0.001 g) to calculate the gut content mass (*W*_G_).

### Amphipod swimming behaviour

Experimental trials to quantify species-specific swimming behaviour and potential predator avoidance behaviour in both amphipod species were performed within 30 min intervals. For each amphipod species, 40 individuals were tested per trial with five replicates each. Gobies (n = 10) were not fed 24 h prior to the experiments. Amphipods transferred to the test aquaria were allowed to settle and shelter for 1 h before start of the trial. A single trial consisted of 15 min without plus 15 min with a single *N. melanostomus* present in the aquarium. A photograph was taken every minute to enable standardised counting of amphipods swimming freely in the water column. Experimental trials were conducted with substratum provided in the setup. Since amphipod species could not be differentiated exactly when observing them from outside the tanks, this experiment was not conducted for a mixed species setup.

### Data analysis

To quantitatively test for differences in the overall feeding between *N. melanostomus* sexes within the experimental timeframe, the index of stomach fullness (ISF) = 100 *W*_G_ *W_T_^−1^ was calculated, providing a standardized and representative estimate of prey consumption [7]. The general assumption was tested that females have a higher feed-uptake compared with males, due to a higher energy demand and potential differences in *W*_T_ due to variable fish fecundity during the reproductive state in the respective time of the year. Since also single fish and amphipod biomasses were not identical in the experiments, the use of ISF (instead of amphipod counts) to quantify prey consumption enabled higher statistical power, since extremes could be included. For the comparison of differences in feeding on different species and size classes of amphipods, consumption ratios were used as dataset.

Selectivity of goby predation was evaluated using Manly’s selectivity index α (discussed by Chesson [33]), calculated according to Eq. 1:

1 $$\alpha_{i} = \frac{{\ln (\frac{{n_{i0 } - r_{i} }}{{n_{i0} }})}}{{\mathop { \Sigma }\nolimits_{j = 1}^{m} \ln (n_{j0} - r_{j} )/n_{j0} }} ,\quad i = 1,2, \ldots , m$$

where *n*_*i0*_ is the initial number of prey species *i*, *r*_*i*_ is the number of prey species *i* consumed by the goby and m is the number of species, which is two in this study.

In some cases all individuals of the preferred prey species were consumed in the trial, so calculation of α was not possible. To account for that, the approach by Klecka and Boukal [34] was applied, modifying the equation in single cases. Therefore one additional individual was added to the respective *n*_*i0*_ and *n*_*j0*_, assuming this additional individual would have survived. This was the case for two trials in which all *G. pulex* were consumed, resulting in a slightly conservative estimate of α. Dead individuals were excluded from this calculation. Statistical analysis were performed on Manly’s alpha (α). Differences in selective feeding of the fish were tested by comparing calculated Manly’s α values against a hypothetical value of 0.5 (no selectivity) using Wilcoxon’s one sample test. For graphical presentation, the alpha values were converted in electivity indices as described in Chesson [33].

2 $$\varepsilon_{i} \,= \frac{{m\alpha_{i} - 1}}{{\left( {m - 2} \right)\alpha + 1}},\quad i = 1, \ldots ,m,$$

Electivity index ε can reach values between −1 and 1 per prey species, where positive values indicate a preferred prey and a value of 0 corresponding to unselective feeding.

All datasets were tested for normal distribution and homogeneity of variance using Shapiro-Wilks and Levene’s test, respectively. For comparison of ISF and sex-specific predation, pairwise t tests were used. As assumptions for parametric tests were not met for comparison of mortality rates in control trials without fish, nonparametric Kruskal–Wallis-test was used for multiple comparisons. For post hoc comparisons, Bonferroni-corrected Mann–Whitney-U tests were used. For comparison of amphipod consumption rate of gobies a factorial ANOVA design was chosen, using the factors species (*G. pulex*, *D. villosus*), substratum (absence, presence) and trial (single, mixed). Since the data was skewed, rank transformation of the data was used to overcome violation of ANOVA assumptions. We applied an aligned rank transformation procedure by Wobbrock et al. [35], which is suitable for factorial designs including interactions, since the same main effect, and interaction structure as the original data is retained. Analyses were conducted on partial datasets since including single species and mixed species trials in the same model was not possible, due to the non-independent data. Species were analysed separately using a between subject design with the factors substratum (absence, presence) and trial (single, mixed). *Gammarus pulex* and *D. villosus* were compared with each other separately in single species trials, using a between-subject-design with the factors species (*G. pulex*, *D. villosus*) and substratum (absence, presence). Subsequently, one-way ANOVAs were conducted to compare differences in single vs. mixed trials with and without substratum separately. Mixed species trials for both same and different size-class comparisons were tested using a within-subject-design (repeated measures ANOVA) and are presented separately for each size class comparison.

To test for differences in swimming activity and potential predator avoidance of amphipods, Wilcoxon signed-rank test was applied to compare numbers of free-swimming gammarids with and without fish present (average of 15 observations in 5 tanks). Statistical analyses were performed using the software SPSS 22 (IBM, USA). Significance was accepted at p < 0.05.

## Results

### Amphipod mortality without predator

No significant predation effects (either asymmetric predation by IGP or symmetric by cannibalism) between the tested amphipod species were observed in the trials with the same size class (size class 1) of amphipods, within the test duration of 3 h. Control trials without predator presence revealed similar mortalities in both *G. pulex* and *D. villosus* which remained on average below 2 % (Table 2A). No significant differences were found neither between trials with and without substratum (single trials, Mann–Whitney: *G. pulex*, z = −0.83, p > 0.05; *D. villosus*, z = −0.27, p > 0.05; mixed trials, Mann–Whitney: *G. pulex,* z = −1.76, p > 0.05; *D. villosus*, z = −2.46, p > 0.05), nor between single- or mixed-species trials (without substratum, Mann–Whitney: *G. pulex*, z = −1.78, *D. villosus*, z = −2.93, p > 0.05; with substratum, Mann–Whitney: *G. pulex,* z = −0.94, p > 0.05; *D. villosus*, z = −0.67, p > 0.05). Control trials with different size classes of the two amphipod species (e.g. *G. pulex* size class 1, *D. villosus* size class 2 resulted in different mortality rates in comparison to trials with equal size distribution of both species. Higher mortality rates for both species were recorded in the mixed species trial without the presence of substratum (Table 2B), without being statistically significant. Mortality was highest for *G. pulex* (2.5 %) when both amphipod species were held together in the absence of substratum. As above, no significant differences could be found neither between trials with and without substratum (single trials, Mann–Whitney: *D. villosus*, z = −0.18, p > 0.05; mixed trials, Mann–Whitney: *G. pulex,* z = −1.29, p > 0.05; *D. villosus*, z = −1.19, p > 0.05) nor between mixed- or single-species trials (without substratum, Mann–Whitney: *G. pulex*, z = −0.83, *D. villosus*, z = −0.08, p > 0.05; with substratum, Mann–Whitney: *G. pulex,* z = −1.39, p > 0.05; *D. villosus*, z = −1.56, p > 0.05).

**Table 2:** Relative comparison of amphipod losses found after 3 h under experimental conditions without *N. melanostomus* presence

	Trial	Substratum	Mortality [%] mean (± SD)		N
			*G. pulex*	*D. villosus*	
A	Single	Presence	1.8 (±3.0)	1.2 (±2.5)	17
	Mixed	Presence	1.3 (±2.7)	1.3 (±3.2)	24
	Single	Absence	0.9 (±2.0)	1.0 (±2.2)	17
	Mixed	Absence	0.3 (±1.3)	0.0 (±0.0)	29
B	Single	Presence	1.8 (±3.0)	1.7 (±2.1)	6
	Mixed	Presence	0.7 (±1.8)	0.7 (±1.8)	16
	Single	Absence	0.9 (±2.0)	1.3 (±1.4)	6
	Mixed	Absence	2.5 (±4.1)	1.9 (±3.1)	15

*A* *G. pulex* and *D. villosus* with the same size class, *B* *D. villosus* larger than *G. pulex*. *Single* only one species per aquarium, *Mixed* both species in aquarium

### Predation experiments

#### Sex-specific predation

**Fig. 1:**
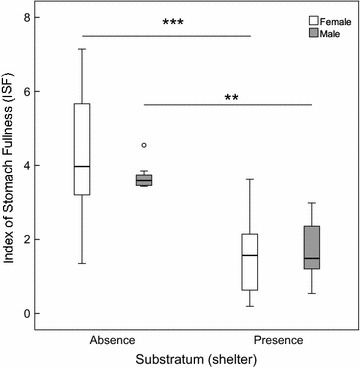
Influence of fish sex on gammarid predation depending on absence or presence of substratum. Index of stomach fullness (ISF) confirms a generally higher predation rate in absence of substratum. No differences between female (*white*) and male (*grey*) *N. melanostomus* were observed. [*Asterisks* indicate significant differences at p < 0.05 (*), p < 0.01 (**), p < 0.001 (***)]. *Boxplots* represent 25 to 75 % (*boxes *) and 5 to 95 % percentiles (*whiskers*). *Circles* represent outliers (exceeding 1.5 interquartile range). n = 14: female/substratum absent; n = 8: male/substratum absent; n = 12: female/substratum present; n = 10: male/substratum present)

Comparing the ISF (Fig. 1), no significant differences in prey consumption between female (mean ± SD, 4.3 ± 1.5) and male (3.3 ± 1.1) fish were observed in experiments, neither without (T_(20)_ = 1.552, p = 0.136) nor with substratum (T_(20)_ = 0.532, p = 0.600). Mean ISF ± SD ranged between 1.50 ± 1.1 for female and 1.52 ± 1.7 for male *N. melanostomus* in presence of substratum. In case of generally reduced availability of amphipods in the open water, i.e. when substratum was provided, ISF was about two times lower than in tests without substratum (Fig. 1). In female *N. melanostomus,* the ISF was significantly (T_(24)_ = 5.259, p < 0.001) lower in treatments with substratum than in treatments without substratum. This finding was also significant (T_(16)_ = 3.538, p = 0.003) in tests with male *N. melanostomus*.

#### Between-subject comparison *Gammarus pulex*

**Fig. 2:**
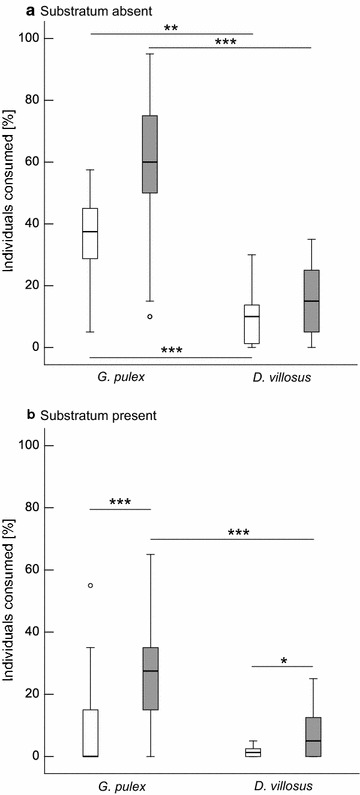
Predation of *N. melanostomus* on amphipods within a 3 h time-period where either single species (*white*, n = 11) or a combination of both species (*grey*, n = 22) were tested. **a** Substratum absent. **b** Substratum present. [*Asterisks* indicate significant differences at p < 0.05 (*), p < 0.01 (**), p < 0.001 (***)]. *Boxplots* represent 25 to 75 % (*boxes*) and 5 to 95 % percentiles (*whiskers*). *Circles* represent outliers (exceeding 1.5 interquartile range)

The main factor substratum had significant effects on the amount of *G. pulex* consumed by *N. melanostomus* (F_(1,100)_ = 12.26, p = 0.001, Table 3). No significant effects of the main factor trial were observed, but a significant interaction between the factors substratum and trial was evident (F_(1,100)_ = 6.03, p = 0.03). Separate examination of the datasets with and without substratum showed that, without substratum, no statistically significant difference was evident between single and mixed trials (Fig. 2). *Neogobius melanostomus* consumed 37 ± 14.7 (mean ± SD) and 58.2 ± 24.9 % of *G. pulex*, respectively. In contrast, a significant difference could be observed in the presence of substratum between single and mixed species trials (F_(1, 69)_ = 22.05, p < 0.001). Gobies consumed 8.6 ± 12.8 (mean ± SD) percent of *G. pulex* in single trials and 27.3 ± 18.2 percent of *G. pulex* when held together with *D. villosus*.

**Table 3:** Comparison of predation rates of *N. melanostomus* on *Gammarus pulex* based on factorial ANOVA with the factors trial (single species vs. mixed species) and substratum (absence vs. presence)

Factor	*SS*	*df*	*MS*	*F*	*p*
Trial					
	7284.869	1	7284.869	2.565	0.112
Error	283960.694	100	2839.607		
Substratum					
	29647.329	1	29647.329	12.256	*0.001*
Error	241893.087	100	2418.931		
Interaction trial x substratum					
	20296.829	1	20296.829	6.031	*0.016*
Error	336567.269	100	3365.673		

Italic values indicate significance of p value (p < 0.05)

*SS* sum of squares, *df* degrees of freedom, *MS* mean square, *F* F value, *p* p value of single factors and their interactions, respectively

#### Between-subject comparison *Dikerogammarus villosus*

Similar to the results of *G. pulex*, strongest effects on *D. villosus* predation were observed for the main factor substratum (F_(1,65)_ = 22.97, p < 0.001; Table 4). Also the main factor trial showed to be significant (F_(1,65)_ = 6.88, p = 0.01), but no significant interaction between the main factors was observed. Separate examination of the datasets without and with substratum showed that, without substratum there were no significant differences in consumed *D. villosus* from single and mixed trials (Fig. 2). *N. melanostomus* consumed 9.5 ± 9.3 (mean ± SD) and 16.4 ± 11.7 percent of *D. villosus*, respectively. In the presence of substratum as shelter single and mixed trials were significantly different (F_(1,34)_ = 5.12, p = 0.03). *N. melanostomus* consumed 2.3 ± 3.5 percent of *D. villosus* in single and 6.8 ± 7.6 in mixed trials.

**Table 4:** Comparison of predation rates of *N. melanostomus* on *Dikerogammarus villosus* based on factorial ANOVA with the factors trial (single species vs. mixed species) and substratum (absence vs. presence)

Factor	*SS*	*df*	*MS*	*F*	*p*
Trial					
	20544.067	1	20544.067	6.881	*0.011*
Error	194070.532	65	2985.7		
Substratum					
	61621.819	1	61621.819	22.974	*<0.001*
Error	174342.766	65	2418.931		
Interaction trial x substratum					
	5549.039	1	5549.039	1.982	0.164
Error	181939.994	65	2799.077		

Italic values indicate significance of p value (p < 0.05)

*SS* sum of squares, *df* degrees of freedom, *MS* mean square, *F* F value, *p* p value of single factors and their interactions, respectively

#### Between-subject comparison—single species trials (*G. pulex* vs. *D. villosus*)

The comparison of predation rates showed that both main factors species and substratum had strong effects of amphipod consumption by *N. melanostomus* (species: F_(1,81)_ = 59.16, p < 0.001; substratum: F_(1,81)_ = 48.91, p < 0.001; Table 5). The observed feeding pattern in *N. melanostomus* revealed a clear preference for *G. pulex* in the experimental trials with amphipods of the same size-class and a higher susceptibility of this native amphipod compared to non-native *D. villosus* to predation in the absence of substratum (Fig. 2). Also a significant interaction of the factors species and substratum was observed (F_(1,81)_ = 7.14, p = 0.009).

**Table 5:** Comparison of predation rates of *N. melanostomus* for single species predation trials based on factorial ANOVA with the factors species (*G. pulex* vs. *D. villosus*) and substratum (absence vs. presence)

Factor	*SS*	*df*	*MS*	*F*	*p*
Species					
	59950.019	1	59950.019	59.165	*p* *<* *0.001*
Error	82074.539	81	1013.266		
Substratum					
	94868.898	1	94868.898	48.913	*p* *<* *0.001*
Error	157103.081	81	1939.544		
Interaction species x substratum					
	12026.53	1	12026.53	7.145	*0.009*
Error	136349.47	81	1683.327		

Italic values indicate significance of p value (p < 0.05)

*SS* sum of squares, *df* degrees of freedom, *MS* mean square, *F* F value, *p* p value of single factors and their interactions, respectively

Separate examination of the datasets with and without substratum showed significant differences between consumed *G. pulex* and *D. villosus* in the absence of substratum (F_(1,20)_ = 12.36, p = 0.002). This was even more pronounced in the presence of substratum (F_(1,61)_ = 60.31, p < 0.001). When *G. pulex* was the only food source available, each goby consumed 15 ± 6 (mean ± SD) amphipod specimens per trial (without substratum) and 4 ± 5 specimens (with substratum present), respectively. If *D. villosus* was the only prey species available, 4 ± 4 (mean ± SD) individuals were consumed without and 1 ± 1 with substratum present. In the latter case, several gobies (21 %) with empty guts were recorded.

#### Within-subject comparison—mixed species trials (*G. pulex* vs. *D. villosus*)

In the mixed species trials, i.e. when both amphipod species were available as prey, round gobies clearly preferred *G. pulex* as prey (Fig. 2). This effect was even more pronounced in the presence of substratum, likely due to biological interactions between the both amphipod species within the shelter (Fig. 2). Predation rates were significantly higher on *G. pulex* (percent consumed: median 47.5, mean 42.7, SD 26.6) compared to *D. villosus* (percent consumed: median 10, mean 11.6, SD 10.9), F_(1,43)_ = 31.34, p < 0.001, with an effect size of 0.42. Separate examination of the datasets with and without substratum showed that without substratum, in 95.4 % cases more *G. pulex* were consumed and there was a significant higher predation on *G. pulex* (percent consumed: median 60, mean 58.2, SD 24.9) compared to *D. villosus* (percent consumed: median 15, mean 16.4, SD 11.7), F_(1,21)_ = 9.5, p = 0.006, with an effect size 0.31). With substratum, in 90.9 % cases more *G. pulex* were consumed and in no case more *D. villosus* were consumed. In 9.1 % of the cases, no difference was found. With substratum, there was a significant higher predation on *G. pulex* (percent consumed: median 27.5, mean 27.3, SD 18.2) compared to *D. villosus* (percent consumed: median 5, mean 6.8, SD 7.6), F_(1,21)_ = 35.24, p < 0.001, and the difference was large (effect size 0.63).

### Size effects

**Fig. 3:**
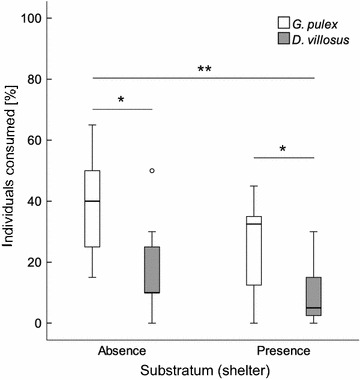
Predation of *N. melanostomus* within a 3 h time-period on *G. pulex* (*grey*) and *D. villosus* (*white*). *Gammarus pulex* mean size (± SD) 11.8 ± 1.5 mm and *D. villosus* 18.3 ± 2.4 mm. [*Asterisks* indicate significant differences at p < 0.05 (*), p < 0.01 (**), p < 0.001 (***)]. *Boxplots* represent 25 to 75 % (*boxes*) and 5 to 95 % percentiles (*whiskers*). *Circles* represent outliers (exceeding 1.5 interquartile range); n = 12 per substratum trial)

In the mixed trials with larger *D. villosus* (size class 2) vs. smaller *G. pulex* (size class 1), similar trends were observed as with equally sized amphipods (Fig. 3). Significantly more *G. pulex* were consumed as compared to *D. villosus*, regardless if substratum was absent (F_(1,10)_ = 8.44, p = 0.16, effect size 0.46) or present (F_(1,11)_ = 24.68, p < 0.001, effect size 0.69). Again, the availability of substratum as shelter resulted in a stronger effect on predation. The fish consumed more *G. pulex* (percent consumed: median 40, mean 38.2, SD 16.9) than *D. villosus* (percent consumed: median 10, mean 16.8, SD 14.7) in 83.3 % of the cases in the absence of shelter. With substratum provided, more *G. pulex* (percent consumed: median 32.5, mean 26.2, SD 14.8) than *D. villosus* (percent consumed: median 5, mean 8.7, SD 8.8) were consumed in 90.9 % of the cases.

### Selectivity index

**Fig. 4:**
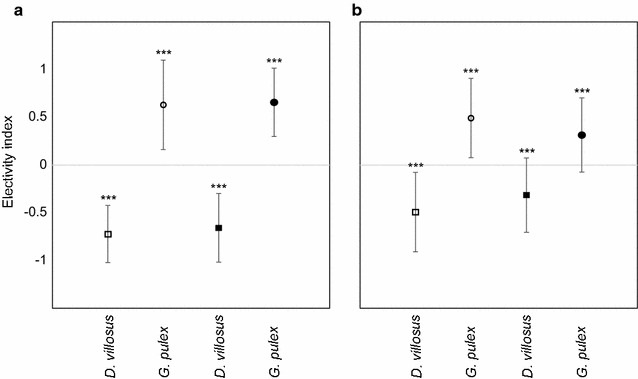
Prey selectivity of *N. melanostomus*. Mean values ± SE of electivity index are presented. Positive values represent preferred prey species in presence (*open circles* and *squares*) or absence (*black circles* and *squares*) of substratum. *Dashed line* indicates no selectivity. **a** Same size class of *G. pulex* and *D. villosus*. **b** Larger size class of *D. villosus*. Asterisks indicate significant differences to zero at p < 0.05 (repeated measures ANOVA): (*), p < 0.01 (**), p < 0.001 (***)

Selectivity analyses using Manly’s alpha confirmed a clear preference for *G. pulex* as prey item in mixed species trials (Fig. 4). Alpha values above 0.5 indicate a preference for *G. pulex* and were highest in trials without substratum added and the same size-class of the two amphipod species (0.83 ± 0.18, mean ± SD) compared to trials with presence of substratum (0.81 ± 0.23, mean ± SD). Selectivity was significant for *G. pulex* as preferred prey item (without substratum: z = 3.94, p < 0.001; with substratum z = 3.49, p < 0.001). Interestingly, no significant preference was evident from the selectivity index observed for in the different size-class trials, but higher values in the presence of substratum (0.65 ± 0.37, mean ± SD) were observed compared to trials without substratum (0.50 ± 0.38, mean ± SD).

### Amphipod swimming behaviour

**Fig. 5:**
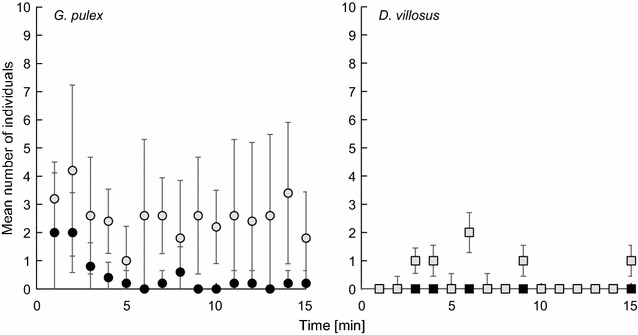
Mean number (± SD) of individual amphipods swimming actively in the water column within a timeframe of 15 min in absence (*grey*) and 15 min in presence (*black*) of a single *N. melanostomus* specimen. (n = 5 per species)

Generally, behaviour of the amphipods differed significantly between species: *Gammarus pulex* was about three to four times more active in terms of individuals swimming freely in the water-column during the observation period (Fig. 5). After adding the predator, there was a significant reduction in freely swimming *G. pulex* (mean 2.53, SD 1.67 per min) compared to controls without gobies (mean 0.46, SD 0.27 per min, n = 5), z = −2.02, p = 0.042, and the difference was large (effect size r = −0.90).

*Dikerogammarus villosus* showed a generally low activity, even in the control group (mean 0.32, SD 0.05 per min, n = 5). After addition of the predator, this effect became more pronounced (mean 0.01, SD 0.03). Wilcoxon comparison revealed a significant difference in activity patterns caused by the presence of the fish (z = −2.03, p = 0.042, effect size r = −0.91). Pairwise comparison between species identified significant differences between the control groups (z = −2.63, p = 0.009, effect size r = −0.83) and in the presence of the fish (z = −2.12, p = 0.034, effect size r = −0.67).

## Discussion

In several European waterbodies, the arrival of non-native *D. villosus* has simultaneous occurred as the disappearance of native amphipods [16, 36, 37] which is often explained by asymmetric mutual predation [24]. A sympatric and synergistic impact by invasive *D. villosus* and invasive *N. melanostomus* has not yet been considered, but appears likely based on the results of our study.

In our experiments, *D. villosus* faced a lower predation risk against *N. melanostomus* due to a relatively lower swimming activity as compared to *G. pulex*. These findings match obervations in other species pairs such as those by Kinzler and Maier [23] who detected similar results for rainbow trout and European perch. According to van Riel et al. [38], the presence of *D. villosus* can lead to an increased number of *G. pulex* swimming freely in the water column, indicating interference competition. Analogously, we observed a threefold higher predation of *N. melanostomus* on *G. pulex* in the presence of *D. villosus* when shelter was available compared to a single exposure of *G. pulex*. Since substrate structures are often heavily modified in natural habitats for bank erosion protection, this may directly affect predation risk of native gammarids, particularly in anthropogenically modified habitat structures.

### The role of IGP

*Dikerogammarus villosus* is able to prey on other amphipod species without being restricted to vulnerable post-molting stages [36]. On the other hand, the mouthparts of *D. villosus* are not highly specialized just for carnivory and predation, and the species has been described to use a wide spectrum of different food sources [39, 40]. Also gut contents and stable isotope analyses by Koester and Gergs [41] did not provide evidence for *D. villosus* being a carnivorous “killer shrimp”. In our study, no significant asymmetric mutual predation between both amphipod species could be confirmed within the short experimental duration. Gammarids are also known to naturally occur at similar densities and the selected experimental setup allowed for isolated analysis of the effects caused by *N. melanostomus* predation. Based upon the results of our experiments, intraguild predation seemingly only plays a minor role and cannot explain the invasion success of *D. villosus* outside of its natural distribution range. In contrast, interactions with *D. villosus* seem to facilitate excessive consumption of the native gammarid by the newly introduced top-predator *N. melanostomus* and appears to play a more important role in the decline of autochthonous amphipods.

Structural diversity of invaded habitats can mitigate potential impact by spatial separation due to different habitat preferences, since it uncouples competition between native and invasive species [42]. Especially in heavily modified waterbodies such as shipping canals, structural diversity is much lower compared to natural aquatic systems. Technolithal and other monotonic structures within these waterbodies can thus force the respective species towards direct interaction and may consequently increase competitive stress in autochthonous, less generalized species.

Thus, main behavioural traits acting in combination can provide a plausible explanation for the observed eradication of *G. pulex* in natural habitats invaded by *D. villosus* and *N. melanostomus*. In case of *N. melanostomus* and *G. pulex,* preference for one species as a superior food item accompanied by higher susceptibility to predation by different activity patterns in swimming behaviour interacts synergistically and speeds up this process.

Comparing predation of *N. melanostomus* against *G. fasciatus,* an autochthonous amphipod species native to North America, and its Ponto-Caspian invasive counterpart *E. ischnus*, Kestrup and Ricciardi [43] found that competition with *E. ischnus* did not increase the vulnerability of *G. fasciatus* to goby predation. In their experiments *E. ischnus* and *G. fasciatus* appeared to be equally susceptible to goby predation and no preference in feeding of *N. melanostomus* was observed. Kestrup and Ricciardi [43] finally concluded that *N. melanostomus* did not influence the replacement of *G. fasciatus* by *E. ischnus* in the St. Lawrence River. Remarkably, outside laboratory conditions in Lake Erie and Lake Ontario, where *N. melanostomus* continues to spread, *E. ischnus* has replaced *G. fasciatus* as the dominant amphipod on substrates fouled by dreissenids [44]. These amphipods are mutual predators, too, and dominance of competing amphipods varies with conductivity [45]. Thus, IGP can also be influenced by environmental heterogeneity.

Since *N. melanostomus* used in Kestrup and Ricciardi [43] had a significantly lower (mean = 6.6 cm ± 0.1 SE) *L*_T_ compared with our experiments, the experimental setup chosen might have led to misinterpretation: Due to a significantly smaller gape limitation, *N. melanostomus* of this size class preliminary feeds on smaller prey than adult amphipods, such as chelicorophiids, isopods, zooplankton and juvenile amphipods [7]. At a size of about 10–12 cm, depending on time since invasion, *N. melanostomus* is known to mainly feed on amphipods correlating with an ontogenetic diet switch [8]. Thus, in our experiments *N. melanostomus* and amphipods were size-selected with expected maximum impact.

### Selective feeding by fish or different vulnerability

Among others, predation by fish is an important factor influencing the composition of amphipod communities. *Neogobius melanostomus* has a rather opportunistic feeding strategy, using the most available prey as compared to other more specialized goby species such as *P. kessleri* [7, 29]. However, *N. melanostomus* shows a clear preference for amphipods over molluscs at early stages of the invasion process when their availability is not limited [8]. Thus, according to our experiments, amphipods appear to be a superior and favourite prey for both sexes, likely facilitating the invasion success of invasive gobies [7, 28, 46]. The choice of amphipods as important prey item in our study appears legitimate since the proportion of amphipods in *N. melanostomus* prey is highest in all seasons: about two thirds to three quarters to the total food uptake and amphipods were consumed in higher proportions as compared to their availability in environment [7]. Other potentially preferred prey items such as Chironomid larvae have limited availability due to emergence of imagos in summer and autumn. Amphipods can percept predatory fish or injured conspecifics by chemical stimuli and are thus able to react by predator avoidance behaviour (co-evolutionary adaptation) such as reducing time in open water [47], or change of habitat use [48]. Behavioural responses can even be flexible when complex microhabitat structures are available [49]. Since amphipods are known to possess distinct substrate preferences, the presence of preferred microhabitats can mitigate their predation risk [24]. *Dikerogammarus villosus* preferred gravel with a low percentage of sand and stones, whereas *G. pulex* showed no clear substratum preference in a substratum choice experiment [38]. However, in line with our study (see Fig. 5), in presence of invasive *D. villosus*, native *G. pulex* can be found significantly less frequent in gravel [50, 51] independent of *D. villosus* abundance [38, 41]. Such an exclusion from shelter-providing habitats can lead to increased swimming activity in *G. pulex* and consequently to an increased exposure to predatory fish. It might therefore play an important role in the displacement of native amphipods by *D. villosus* [51]. Besides the feeding behaviour, high reproductive potential and rapid growth of *D. villosus* [16, 52, 53] make this species a strong competitor not only for food but also for habitat space [41]. In our study, both sexes of invasive *N.melanostomus* asymmetrically consumed significantly more native *G. pulex* than invasive *D. villosus*, regardless if there was substratum present or not. Calculations of a preferential prey choice index [34] corroborated these findings. On a first glance, these findings are in contrast to Kley et al. [24], who considered that differences in use of spatial niches can permit the co-existence of *D. villosus* and *G. roeseli* in the wild when substrates are diverse. However in their tests, it was autochthonous burbot (*Lota lota*) that did not affect substrate choice or predation risk of *G. roeseli* in presence of *D. villosus*. It is probably due to the smaller prey selectivity of *L. lota* compared to the invasive *N. melanostomus* which can explain these differences. For freshwater amphipods, a predator avoiding mechanism would be to respond to an open water predator by reducing activity and moving towards the sediment, whereas a benthic predator would generally be better avoided by an active escapement through swimming or increased drift escape when predator abundance is low [47, 49]. In case of high or very high numbers of predators, most likely after mass development of invasive alien species, hiding seems to be a better survival strategy. Proportion of invasive *N. melanostomus* to the total fish fauna can be extremely high, particularly in man-made rip rap habitats where they have been reported to contribute two thirds of all fish counts and about 50 % of total fish biomass [9]. Thus, drift might not work as a perfect escape mechanism. Instead, escaping by drift likely leads to increased predation when predator abundance is high and thus might favour extinction in invaded ecosystems. This theory matches actual field observations from the Danube River, where the breakdown of *G. roeseli* in sections invaded by non-native gobies is reported [7]. Considering these principles, unreduced activity seems to be mostly responsible for higher losses in native *G. pulex*, whereas *D. villosus* appears to be generally less active, consequently being less vulnerable to the benthic ambush predator *N. melanostomus*. According to foraging theories, it therefore appears to be more energy efficient for *N. melanostomus* to feed on native *G. pulex* as an “easy” prey.

The results from our experiments provide a mechanistic explanation for higher feeding rates on *G. pulex* by *N. melanostomus* in absence of suitable substratum which in pristine riverine environments could provide shelter against predation. The observed higher consumption of *G. pulex* by *N. melanostomus* indicates species-specific vulnerability due to gammarid acitivity. Both species similarly respond to a predator, but *D. villosus* is less vulnerable due to its overall lower activity. Consequently, this study provides evidence that a synergistic impact of invasive species with distinct foraging strategies can be greater than the independent effects of the single species. On an ecosystem scale such added effects can pose an important factor in the context of an “invasional meltdown” scenario as defined by Simberloff and Von Holle [30]. Such an invasional meltdown supposedly still seems to occur in the upper Danube River to date [8].

## Conclusions

Behavioural traits and interactions between closely related species are important basic mechanisms in understanding species displacement by IAS. The results of this study suggest that both the foraging selectivity of invasive *N. melanostomus*, as well as the behavioural interaction between *D. villosus* and *G. pulex* together result in an increased predation risk for the native gammarid. Previous invasions of *D. villosus* thus likely facilitate the rapid range expansion of *N. melanostomus*, in turn increasing predation on autochthonous amphipods. The results of this study are thus in line with processes described as “invasional meltdown” on the scale of ecosystems. Not only competition for food and habitat resources as well as asymmetric mutual predation between the closely related (amphipod) species can lead to declines of less competitive autochthonous species, but also becoming an easy prey for a new predator enabling excessive resource consumption may lead to the extinction of autochthonous species. Sympatrically, both invasive Ponto-Caspian IAS possess the ability to effectively restructure food-web composition by mutually and synergistically facilitating their invasive potentials, thus promoting further range expansion of both species.

## Abbreviations

ANOVA: analysis of variance
DO: dissolved oxygen
EC: electric conductivity
IAS: invasive alien species
IGP: intraguild predation
ISF: index of stomach fullness
*L*_T_: total length
SE: standard error
SD: standard deviation
W_T_: total body weight
*W*_G_: gut content weight
